# Congenital Oropharyngeal Teratoma: A Rare Cause of Respiratory and Feeding Difficulties

**DOI:** 10.7759/cureus.98138

**Published:** 2025-11-30

**Authors:** Fleur Tuijl, Fabrice Savaria, Diana Born, Stefan Markart, Sandro Stöckli

**Affiliations:** 1 Department of Otorhinolaryngology, HOCH Health Ostschweiz, St. Gallen, CHE; 2 Department of Pathology, HOCH Health Ostschweiz, St. Gallen, CHE; 3 Department of Radiology, HOCH Health Ostschweiz, St. Gallen, CHE

**Keywords:** airway obstruction, congenital oropharyngeal teratoma, deglutition disorders, infant, oropharyngeal neoplasms, teratoma

## Abstract

Teratomas are rare germ cell tumors composed of mature and/or immature tissues derived from all three germ cell layers. Oropharyngeal teratomas are especially uncommon and can cause life-threatening airway and feeding difficulties. This report presents an 11-month-old female patient with intermittent respiratory noise and early feeding challenges. Imaging revealed a polypoid mass in the left oropharynx, which was surgically excised. Histopathological examination confirmed a mature teratoma. Postoperatively, the patient showed marked improvement in both respiration and feeding, with no signs of recurrence after three months and one year of clinical follow-up. This case highlights the importance of including teratoma in the differential diagnosis of oropharyngeal masses. Early imaging, particularly MRI, is crucial for evaluating tumor composition and extent. Complete surgical resection is critical to prevent recurrence and resolve airway and feeding problems. Although typically benign, pharyngeal teratomas can be life-threatening if undiagnosed or inadequately managed. A multidisciplinary approach integrating pediatrics, otolaryngology, radiology, and pathology is vital for optimal outcomes.

## Introduction

Teratomas are germ cell tumors that contain mature or immature tissues from all three germ cell layers: ectoderm, mesoderm, and endoderm, and therefore exhibit a wide range of tissue types, such as hair, muscle, bone, cartilage, and even more complex organoid structures [1]. Teratomas are more commonly found in the gonads, sacrococcygeal region, mediastinum, and pineal region [1]. Head and neck teratomas account for less than five percent of all teratomas, with oropharyngeal teratomas being exceptionally rare. Their incidence is estimated to range from one in 35,000 to one in 200,000 live births, with a higher prevalence in female births [2]. Oropharyngeal teratomas may be detected prenatally, often associated with a polyhydramnion due to impaired fetal swallowing, or postnatally, where they can cause severe complications such as airway obstruction, dysphagia, and potential interference with critical neurovascular structures [2-4]. Early diagnosis and prompt surgical intervention are critical, as these tumors can pose life-threatening risks to the newborn. A thorough understanding of their embryology, histopathology, and optimal treatment is the key to improving outcome. This case report presents the clinical course, surgical management, and histopathological findings of an 11-month-old female infant with an oropharyngeal teratoma.

## Case presentation

**Figure 1:**
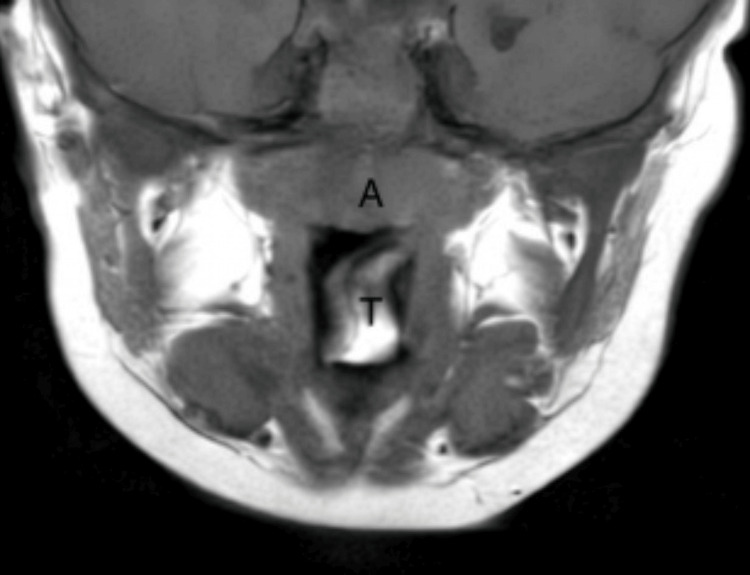
T1-weighted coronal MRI A: Adenoid; T: Teratoma.

An 11-month-old female patient presented in our hospital with a history of intermittent respiratory noise since birth, particularly during inspiration and expiration. The mother reported initial difficulties with feeding, including challenges in breastfeeding and bottle feeding due to prolonged pauses. Improvement was noted after switching to semi-solid foods four months before the consultation. The patient had no episodes of nasal reflux but had a history of intermittent rhinitis associated with infections. There were no previous episodes of otitis media or tonsillitis. On clinical examination, the oral cavity and oropharynx showed a symmetrical palate with a midline uvula and no evidence of cleft formation. A prominent bulge was observed on the left side of the posterior pharyngeal wall. The tonsils were hypertrophic (Grade II bilaterally according to Friedman et al. [5]). During phonation, the palate was elevated symmetrically. The larynx was normal. Nasal examination revealed a midline septum with mild turbinate hypertrophy and adenoid hypertrophy, causing approximately 30% choanal obstruction. A fiberoptic examination confirmed the left-sided bulge adjacent to the left palatine tonsillar arch. Given the clinical presentation with a pharyngeal mass, an MRI was performed. The MRI revealed a 2.2 x 1.5 x 1.2 cm polypoid mass arising from the left palatine tonsil (Figure 1), partially composed of fatty tissue and projecting into the oropharyngeal lumen.

**Figure 2:**
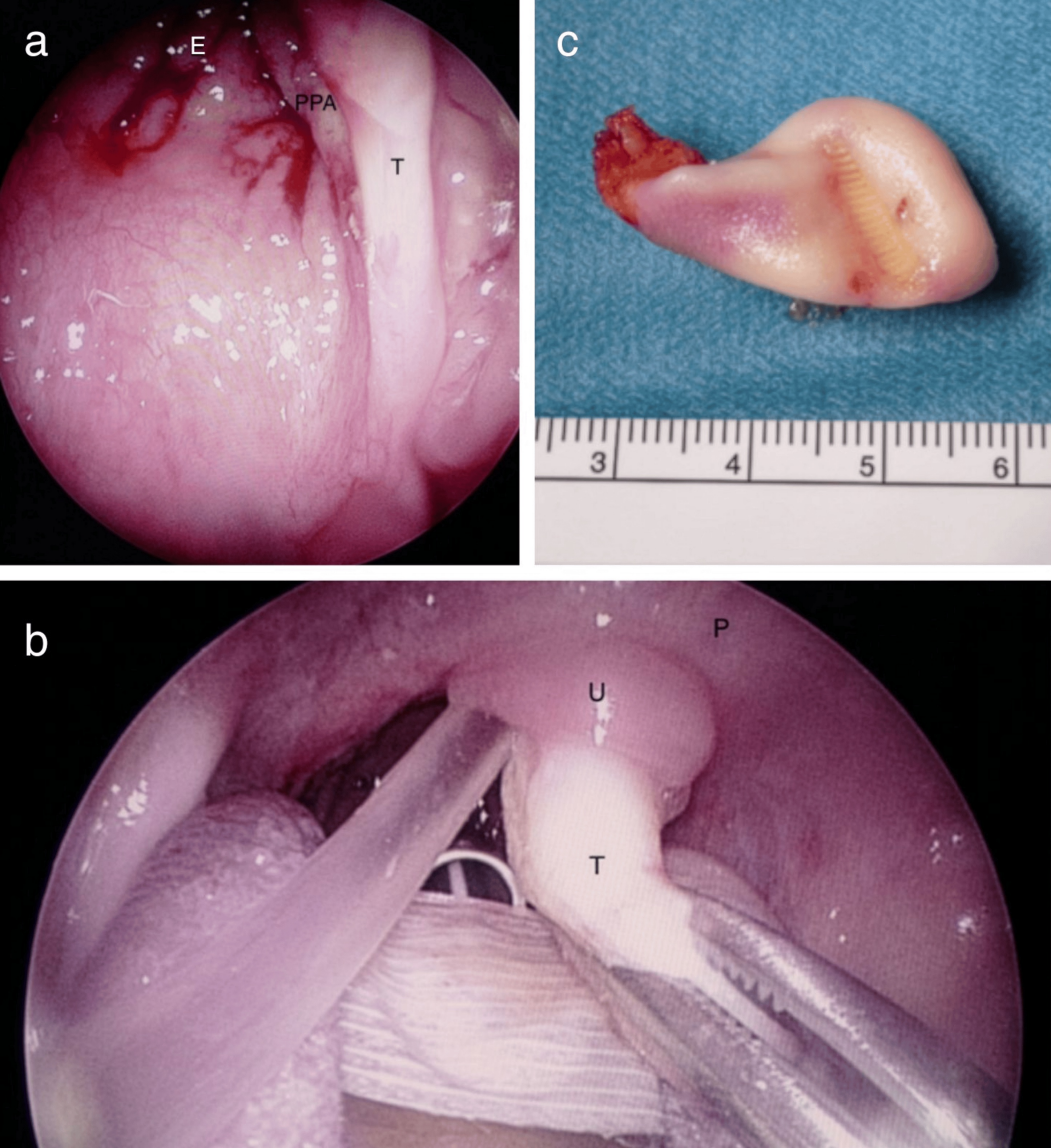
Intraoperative images E: Epipharynx; PPA: Posterior Pharyngeal Arch; T: Teratoma; P: Palate; U: Uvula. (a,b) Oropharyngeal teratoma; (c) Teratoma (cm) after resection.

There was mild medial displacement of the tonsil, but no evidence of additional masses, pathologically enlarged lymph nodes, or abnormal neck soft tissues. Differential diagnosis considered a teratoma or hairy polyp. Excision was performed under general anesthesia. Upon inspection, a pedunculated mass was identified behind the uvula, attached to the posterior pharyngeal wall (Figures 2a, 2b).

The mass was firm upon palpation, pedunculated, and originating from the left posterior tonsillar pillar. The tonsils and the nasopharynx appeared normal. Following bipolar coagulation, the mass was sharply resected at its pedicle using cold steel (Figure 2c).

**Figure 3:**
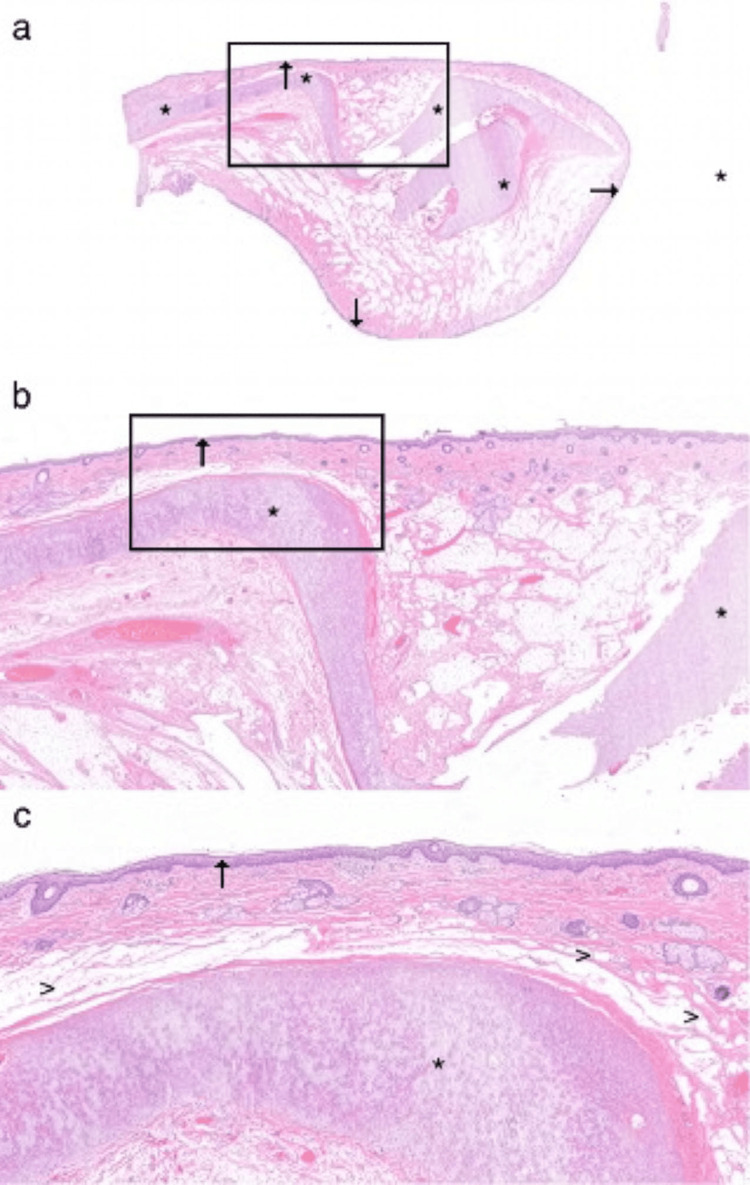
Mature teratoma, hematoxylin and eosin (H&E) staining, showing non-dysplastic squamous epithelium (arrows), cartilaginous structures (asterisks), and mature adipose tissue (arrow heads) Images shown at (a) 0.5x, (b) 1.6x, and (c) 4.5x magnification.

The resected specimen was sent to the pathology department for macroscopic examination. It showed a 2.5 cm polypoid lesion. Histopathology revealed skin appendages, cartilaginous structures, and mature adipose tissue, consistent with a mature teratoma​ below the squamous epithelium (Figure 3).

The complete embedded specimen showed no immaturity and no evidence of malignancy, and was classified as grade 0, according to the Gonzales-Crussi classification [6].

At the three-month follow-up, the patient showed significant improvement in oral intake and overall development. There was no evidence of recurrence, and the patient’s feeding had normalized​. Additionally, at the one-year check-up, there were clinically no signs of recurrence, and the child demonstrated normal development without any dysphagia.

## Discussion

Pharyngeal teratomas pose significant diagnostic and management challenges due to their complex embryological origin, diverse histopathological features, and potentially life-threatening complications. The presented case of an 11-month-old female patient highlights critical aspects of the clinical presentation, surgical management, and long-term outcomes associated with this rare condition.

Clinical presentation 

The clinical manifestation of pharyngeal teratomas varies widely, depending on the tumor’s size, location, and histological composition. In newborns, large masses may cause respiratory distress, stridor, or cyanosis at birth, and often impair feeding, resulting in choking and failure to thrive [1,7-10]. In this case, the infant presented with intermittent respiratory noise and feeding difficulties, consistent with airway compromise commonly reported in the literature. In older children, pharyngeal teratomas might present as nasal obstruction, dysphagia, snoring, or a muffled voice [4]. While prenatal diagnosis is ideal for optimizing perinatal management, many cases, as in this patient, are diagnosed postnatally due to the absence of significant prenatal findings such as a polyhydramnion [3,10-12].

Embryological, pathological, and diagnostic Insights

It is believed that teratomas arise from totipotent germ cells displaced during embryogenesis. In the head and neck region, these cells may originate from the primitive streak or the region of Rathke’s pouch, as suggested by various embryological theories [1,9,12]. 

The complex germ cell layer lesions of the naso-oropharynx are classified by Arnold in 1870 into four distinct histological groups: dermoids, teratoids, teratomas, and epignathi. Dermoids (e.g., “hairy polyps”) contain only ectodermal and mesodermal cells. Teratoids are poorly differentiated, immature tumors that include cells from all three germ cell layers but are loosely organized. True teratomas, like in this case, contain mature tissue from all three germ cell layers and show greater structural organization. Epignathi likewise involve all three germ cell layers; however, they exhibit a high degree of tissue maturity, often showing organoid differentiation, such as teeth, osseous tissue, and hair [13]. 

Currently, there are few reliable prenatal markers to predict outcomes. A polyhydramnion (an abnormal increase in amniotic fluid) remains the most consistent prenatal indication of a oropharyngeal teratoma. The most reliable diagnostic tool is MRI, from which the tracheoesophageal index (reflecting the degree to which the tracheoesophageal complex is displaced from its usual position in front of the cervical spine), can help assess the likelihood of respiratory difficulties in the newborn. Both polyhydramnios and a high tracheoesophageal index are associated with a greater risk of respiratory complications at birth [14]. 

Alpha-fetoprotein (AFP) and beta-human chorionic gonadotropin (β-hCG) serve primarily as surveillance markers for recurrent germ cell tumors (e.g., teratomas), rather than for initial diagnosis. Because newborn AFP levels, especially in premature infants, may be innately high, accurate interpretation can be challenging. Elevated levels of these markers can suggest tumor relapse. However, persistent elevation, often linked to incomplete tumor excision, further complicates how these markers should be interpreted [1,15].

Imaging studies, particularly MRI, play a pivotal role in the diagnosis and surgical planning of pharyngeal teratomas. The MRI findings in this case demonstrated a polypoid mass with fatty tissue, consistent with teratomas’ characteristic heterogeneous composition [3,16,17]. MRI studies allow a detailed assessment of tumor composition, extent, and relationship with adjacent structures, facilitating a precise differential diagnosis [7,9,10]. 

While the majority of pharyngeal teratomas are benign, their potential for life-threatening complications necessitates prompt intervention. Rare instances of malignancy, often associated with immature teratoids, highlight the importance of thorough histological examination [9,11,18]. Genetic and molecular analyses may further elucidate the pathways contributing to teratoma formation and behavior [17].

Surgical management

Complete surgical excision remains the cornerstone of treatment for pharyngeal teratomas. The pedunculated resection approach used in this case allowed for safe and complete resection with minimal morbidity [7,11]. Recurrence is generally associated with incomplete primary dissection [19,20]. 

In rare cases of large prenatally diagnosed teratomas, the ex utero intrapartum treatment (EXIT) procedure has emerged as a vital strategy for managing anticipated airway obstruction at delivery. By maintaining placental circulation, the EXIT procedure provides time to secure the airway through intubation or tracheotomy [1,2,8,10]. 

Prognosis and follow-up

Although head and neck teratomas are often a benign disease, they carry a significant risk of intrauterine or neonatal death. Mortality rates may reach up to 37% [21], particularly in cases involving airway obstruction or fetal hydrops. This unfavorable prognosis is largely attributable to their proximity to the airway and vital vascular structures or intrauterine death from fetal hydrops secondary to high output cardiac failure [10,20,22]. 

The prognosis for pharyngeal teratomas is generally favorable, particularly when complete resection is achieved [1,23]. This case highlights significant improvements in feeding, respiration, and overall development following surgery. Long-term follow-up with clinical assessments is essential to monitor for rare instances of recurrence, which are more likely with incomplete resections or immature histological features [11]. 

Future perspectives

Advancements in prenatal imaging and fetal surgery may improve early detection and management of pharyngeal teratomas, potentially reducing perinatal morbidity and mortality. Additionally, molecular and genetic research could offer valuable insights into the pathogenesis of these tumors, leading to more effective targeted therapies and better prognostic outcomes [12]. 

## Conclusions

Although rare, oropharyngeal teratomas should be included in the differential diagnosis of oropharyngeal masses in the newborn and in young children. This case illustrates that even mild symptoms can reflect a significant underlying lesion, underscoring the importance of careful clinical evaluation. Early diagnosis using imaging, particularly MRI, is crucial, as it enables precise characterization of the mass and informs safe surgical planning. Complete surgical resection remains essential to prevent recurrence and to achieve full functional recovery, as demonstrated by the marked postoperative improvement in feeding and respiration. This case reinforces the excellent long-term prognosis generally observed in mature teratomas. Optimal care depends on close collaboration between pediatricians, otolaryngologists, radiologists, anesthesiologists, and pathologists, especially in complex airway cases.
